# Expression, Solubilization, and Refolding Recovery of a Novel l‑Asparaginase–Arginase 1 Chimera from *E. coli* Inclusion Bodies

**DOI:** 10.1021/acsomega.5c12075

**Published:** 2026-03-06

**Authors:** Massiel V. Rivera, Marina Gabriel Fontes, William Henry Roldán, Roberto Carlos Vieira da Silva Junior, Lisandra Herrera Belén, Jorge F. Beltrán, Igor Lopes-Silva, Adalberto Pessoa, Marco A. Stephano, Jorge G. Farias, Tales Alexandre Costa-Silva, Gisele Monteiro

**Affiliations:** † Departamento de Tecnologia Bioquímica-Farmacêutica, Faculdade de Ciências Farmacêuticas, 28133 Universidade de São Paulo, São Paulo 05508-000, Brazil; ‡ Departamento de Ciencias Básicas, Facultad de Ciencias, 28089 Universidad Santo Tomás, Temuco 4780000, Chile; § Departamento de Ingeniería Química, Facultad de Ingeniería y Ciencias, 28057 Universidad de La Frontera, Temuco 4811230, Chile; ∥ Center for Natural and Human Sciences, Federal University of ABC, Santo André, São Paulo 09280-560, Brazil

## Abstract

The preparative expression, purification, and refolding of a novel bifunctional chimeric enzyme, 63_N_-h_C__hARG1, engineered for dual amino acid depletion therapy, are described. A guinea pig–human l-asparaginase hybrid (63_N_-h_C_) was fused to human arginase 1 through a rigid helical linker, and the codon-optimized gene was expressed in *Escherichia coli* BL21­(DE3). Nonclassical inclusion bodies (IBs) were obtained, exhibiting activities of 2.16 ± 0.04 U mL^–1^ for 63_N_-h_C_ and 13.42 ± 0.09 U L^–1^ for hARG1, with a preparative yield of 25.8 ± 0.6 mg mL^–1^ from the lysate. After solubilization in 8 M urea, size-exclusion chromatography and reverse-dilution refolding were performed, restoring activities to 0.22 ± 0.05 U mL^–1^ and 4.66 ± 0.9 U L^–1^, respectively. Structural compatibility and potential dual functionality were supported by in-silico modeling and molecular docking. A scalable workflow for expression optimization and functional recovery of multimeric chimeric proteins from IBs is thus demonstrated, highlighting key parameters governing the refolding of complex fusion proteins and the value of nonclassical IBs as reservoirs for therapeutic enzyme production.

## Graphical abstract

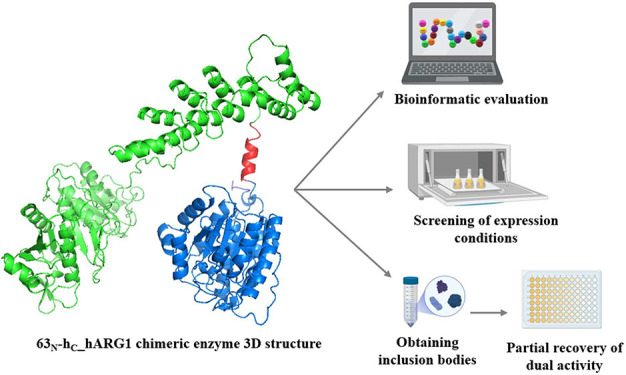

## 1 Introduction

The therapeutic application of amino acid–depleting enzymes has expanded strategies for treating auxotrophic tumors. High catalytic efficiency and molecular specificity are provided by these enzymes, which deplete amino acids and thereby disrupt protein synthesis, leading to impaired tumor proliferation and apoptosis.^1–3^ l-asparaginase (L-ASNase; EC 3.5.1.1) has been widely used in chemotherapy regimens for acute lymphoblastic leukemia (ALL),^4–8^ and its performance has been enhanced through mutagenesis, glycoengineering, and fusion to carrier proteins.^9–11^ The hybrid L-ASNase 63_N_-h_C_, composed of human and guinea pig domains, was developed for improved stability and reduced immunogenicity.^12^ Similarly, human arginase 1 (hARG1; EC 3.5.3.1), which hydrolyzes l-arginine, exhibits antitumor activity in hematologic malignancies such as ALL and AML.^8,13–17^

Engineering chimeric proteins has emerged as a promising approach to enhance performance,^9,18^ especially in cancer therapy, since tumor cells present high plasticity. Functional domains can be combined into a single construct, allowing multiple activities and improved pharmacological properties to be integrated.^19,20^ Chimeric proteins have demonstrated potential in drug delivery, enzyme replacement therapy, and immunotherapy. From a production perspective, dual-activity constructs offer clear advantages: independent expression and purification workflows are reduced, and formulation is simplified. Such streamlining lowers production costs while enhancing scalability and consistency, key considerations in biopharmaceutical manufacturing.^21,22^

Although dual-function chimeric enzymes hold significant therapeutic promise, their recovery from inclusion bodies (IBs) is still not well characterized.

Here, we present a systematic approach for the expression, purification, and refolding of the 63_N_-h_C__hARG1 chimera, focusing on how fusion architecture, oligomerization constraints, and refolding conditions shape the recovery of domain-specific activities. In particular, we address the intrinsic refolding difficulty imposed by combining a tetrameric^12^ l-asparaginase domain with a trimeric^57^ hARG1 domain within a single polypeptide chain, which creates incompatible quaternary assembly requirements and increases the propensity for misfolding and aggregation. The chimera was expressed in *E. coli* BL21­(DE3) and accumulated mainly as IBs. Functional recovery was achieved through a workflow of IB isolation, solubilization, chromatographic purification, and systematic refolding, with efficiency and enzymatic activity assessed.^23–25^ These findings provide insights into scalable production of dual-function chimeric enzymes and demonstrate how fusion design impacts activity and process efficiency.

## 2 Materials and Methods

### 2.1 Construction of Expression Vectors

The coding sequence for 63_N_-h_C_ hybrid,^12^ and hARG1 (UniProt ID: P05089) were fused through a rigid helix-forming linker A­(EAAAK)_2_A,^26,27^ generating the bifunctional construct 63_N_-h_C__hARG1. A codon-optimized synthetic gene was synthesized (GenScript, NJ, USA) and cloned into pET-22b­(+) via *NcoI/HindIII* sites, resulting in a protein with an N-terminal *pelB* leader sequence (Figure 1a). The complete amino acid sequence is provided in Supplementary Text S1. To facilitate downstream purification, a His-tagged version was subcloned into pET-28a­(+) using *NcoI/XhoI* restriction sites. The resulting construct (Figure 1b) was confirmed by colony PCR, verified by Sanger sequencing, and maintained in *E. coli* DH5α.

**Figure 1:**
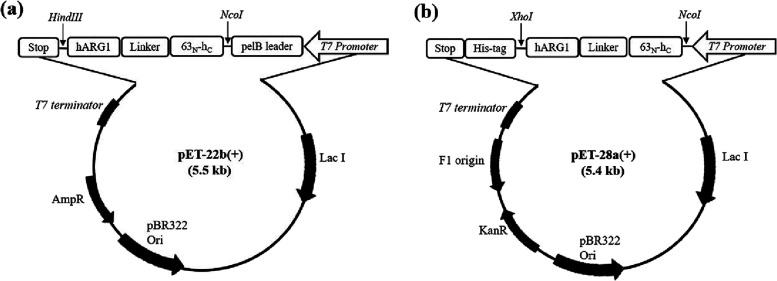
Schematic representation of the expression vectors used in this study. (a) The recombinant vector pET-22b­(+) for the expression of the 63_N_-h_C__hARG1 chimera in *E. coli*. (b) The recombinant vector pET-28a­(+) was used for expression of the chimera in *E. coli*, incorporating a C-terminal His-tag. The 63_N_-h_C__hARG1 gene was amplified from the 63_N_-h_C__hARG1/pET-22b­(+) vector.

### 2.2 Screening of Expression Conditions

The 63_N_-h_C__hARG1/pET-22b­(+) plasmid was transformed into several *E. coli* strains (*AD494, ArcticExpress (DE3), BL21­(DE3), CodonPlus-RIPL (DE3), C43­(DE3), Origami (DE3), Rosetta (DE3), and Tuner (DE3)*) to assess expression efficiency (strain features in Supplementary Table S1). Cultures were grown in lysogeny broth (LB) medium supplemented with 50 μg mL^–1^ of carbenicillin and appropriate antibiotics, when necessary, to mid log phase (OD_600_ nm ≈ 0.8). Expression was induced with Isopropyl β-D-1-thiogalactopyranoside (IPTG) (INLAB, SP, Brazil) (1 mM) and carried out at 37 °C for 3 h. For *ArcticExpress (DE3)*, expression induction was performed at 11 °C for 16 h, as per the manufacturer’s guidelines. After induction, cells were harvested, lysed with BugBuster reagent (Merck, Burlington, MA, USA), and fractionated into soluble and insoluble protein pools. Both fractions were analyzed by SDS-PAGE and enzymatic activity assays to evaluate strain performance and expression profiles. To optimize expression across *E. coli* strains, IPTG was tested at concentrations from 0.01 to 1 mM, and postinduction temperatures were varied (11–20 °C for *ArcticExpress* and 20–37 °C for the other strains). In all cases, cultures were induced at OD_600_ nm ≈ 0.8 and incubated for 16 h.

### 2.3 Isolation of IBs

Protein expression was performed in 1 L LB cultures under optimized conditions (1 mM IPTG, 3 h, 37 °C). Cells were harvested, and resuspended in lysis buffer (50 mM Tris-HCl, pH 7.4; 100 mM NaCl; 1 mM EDTA; 1 mM PMSF; 7 mM 2-β-mercaptoethanol) containing 0.5 mg mL^–1^ egg white lysozyme (Sigma-Aldrich, Missouri, USA) at 10 mL/g of wet cell pellet. After 2h incubation, the suspension was sonicated on ice to prevent heating (30% amplitude, 6 cycles of 10 s on/30 s off), the lysate was centrifuged (13,000×*g*, 20 min, 4 °C), and the pellet was sequentially washed with 0.5% (v/v) Triton X-100-containing and detergent-free buffers, followed by water. The resulting IBs were resuspended in Tris buffer (50 mM, pH 7.4) and analyzed for protein content by BCA assay (Thermo Scientific, Massachusetts, USA), SDS-PAGE, and enzymatic activity. Protein quantification was performed after 2% (w/v) SDS solubilization, ensuring near-complete denaturation.^28^

### 2.4 Solubilization of IBs and Purification by Gel Filtration

The solubilization protocol was adapted from Singh et al.^25^ Isolated IBs were quantified and diluted to ∼1 mg mL^–1^ in solubilization buffer (50 mM Tris-HCl, pH 7.4; 100 mM NaCl; 7 mM 2-β-mercaptoethanol) supplemented with different denaturants: 2, 4, and 8 M urea, 3 and 6 M guanidine hydrochloride (GdnHCl), 2% (w/v) SDS as a positive control, or buffer without denaturant as a negative control. Samples were incubated at 25 °C for 1 h with agitation, followed by centrifugation (15,000×*g*, 30 min, 4 °C) and filtration (0.45 μm). Supernatants were analyzed by BCA assay. A subsequent precipitation step with 100% ethanol was performed to eliminate residual denaturants prior to SDS-PAGE analysis and accurate protein quantification.^29^ For preparative purification, solubilized IBs (∼9 mg mL^–1^) were subjected to size-exclusion chromatography on a Superose 6 Increase 10/300 GL column (Cytiva, Marlborough, USA) using an ÄKTA Purifier system. Runs were performed at 0.2 mL min^–1^ at room temperature, and 1 mL fractions were collected. Fractions containing the target chimera, confirmed by SDS-PAGE, were pooled and stored at 4 °C for subsequent refolding optimization.

### 2.5 Screening of Renaturation Additives and Protein Refolding

Refolding conditions were optimized following Burgess^24^ to enhance efficiency and reduce aggregation. Solubilized protein (∼1 mg mL^–1^) was diluted in refolding buffer (50 mM Tris-HCl, pH 7.4; 100 mM NaCl; 7 mM 2-mercaptoethanol) containing 0.5 M urea or GdnHCl and one additive (125 mM dextran, 200 mM fructose, 100 mM glutamic acid, 2% sucrose, or 10% glycerol). Mixtures were gently stirred and incubated at room temperature for 1 h, and turbidity was monitored at OD_320_ nm. Refolding was performed by dropwise dilution or stepwise dialysis. For dilution, 125 μL protein was added to 2.5 mL buffer at 0.1 mL min^–1^, incubated 1 h at 25 °C, centrifuged (16,000×*g*, 20 min), filtered (0.45 μm), concentrated, and desalted by ultrafiltration (Amicon Ultra 10 kDa, Merck). For dialysis, 10 mL protein (∼1 mg mL^–1^) was dialyzed against 0.5 L refolding buffer described above, at 4 °C for 4 h with three buffer changes. Pooled refolded samples were stored for activity assays.

### 2.6 On-Column Refolding and Purification of 63_N_-h_C__hARG1-His-tag

An overnight culture of *E. coli* *BL21­(DE3)* carrying 63_N_-h_C_ _hARG1/pET-28a­(+) was used to inoculate 1 L of LB with 50 μg mL^–1^ kanamycin. Cultures were grown to OD_600_ nm ≈ 0.8 and induced as described. Cells were harvested (4,000×*g*, 20 min, 4 °C), and IBs were isolated using lysis buffer (50 mM Tris-HCl, pH 7.4; 100 mM NaCl; 20 mM imidazole; 1 mM PMSF; 7 mM 2-β-mercaptoethanol). IBs (50 mg/10 mL) were solubilized in buffer containing 8 M urea, incubated 1 h at 25 °C with shaking, and vortexed every 15 min. After centrifugation (15,000×*g*, 30 min) and filtration (0.45 μm), denatured protein was loaded (1 mL min^–1^) onto a 5 mL Ni^2+^-charged HiTrap IMAC column (Cytiva) pre-equilibrated. The column was washed with 50 mL of buffer containing 50 mM imidazole and 5% glycerol. On-column refolding was performed on an ÄKTA Purifier system using a linear gradient from 8 to 0 M urea in refolding buffer (50 mM Tris-HCl, pH 7.4; 100 mM NaCl; 20 mM imidazole; 5% glycerol; 7 mM 2-β-mercaptoethanol) at 0.1 mL min^–1^ over 50 mL. Refolded protein was eluted with refolding buffer containing 500 mM imidazole. Fractions were collected every 1 mL, pooled, and desalted using a PD-10 Sephadex G-25 column (Cytiva).

### 2.7 Enzyme Volumetric Activity Assays

L-ASNase activity (U mL^–1^) was determined using a microplate-adapted Nesslerization assay with l-asparagine as the substrate, and ammonia release was quantified at 436 nm.^30^ A blank sample was prepared under identical conditions, except that the protein was added after trichloroacetic acid (TCA) addition. One unit (U) of l-asparaginase activity was defined as the amount of enzyme that releases 1 μmol of ammonia per minute at 37 °C. hARG1 activity (U L^–1^) was measured using a commercial assay kit (MAK112, Sigma-Aldrich) based on urea formation, quantified at 430 nm. The blank was prepared under the same conditions, except that the substrate was added after the buffer/urea detection reagent. One unit (U) of arginase 1 activity corresponds to the formation of 1 μmol of urea per minute at 37 °C and pH 9.5. Specific activities (U mg^–1^) were calculated by normalizing volumetric activities to protein concentrations determined by the BCA assay.

### 2.8 SDS-PAGE and Western Blotting Analyses

Protein expression and purification were assessed by SDS-PAGE (10%). Samples were combined with DTT and 5× loading buffer, heated at 95 °C for 5 min, and electrophoresed at 180 V for 80 min in Tris–Glycine buffer. Gels were stained with 0.1% (w/v) Coomassie Brilliant Blue R-250, followed by destaining for 16 h. Detection of the His-tagged chimera was carried out by Western blotting. Proteins were transferred to PVDF membranes using a semidry system (Bio-Rad, CA, USA) at 22 V for 1 h in Towbin buffer (25 mM Tris, pH 8.3; 192 mM glycine; 10% (v/v) methanol). Membranes were blocked with 5% (w/v) skim milk in T-TBS for 2 h, washed, and incubated for 2 h with anti-His tag (C-terminal) antibody conjugated to alkaline phosphatase (1:1,500; Invitrogen, CA, USA) in T-TBS containing 0.1% (v/v) Tween-20 and 1% BSA. Detection was achieved using BCIP/NBT chromogenic substrates.

### 2.9 Bioinformatic Analyses

Physicochemical properties of the chimeric protein were calculated using ProtParam tool (https://web.expasy.org/protparam/). Solubility was predicted with Protein-Sol (https://protein-sol.manchester.ac.uk/), and secondary structure with GOR IV algorithm (https://npsa-prabi.ibcp.fr/cgi-bin/npsa_automat.pl?page=/NPSA/npsa_gor4.html). Disulfide bonds were assessed using SCRATCH server (https://scratch.proteomics.ics.uci.edu/). The monomer structure was predicted with AlphaFold2 ^31^ using the top pLDDT model. Quality was checked with MolProbity (https://molprobity.biochem.duke.edu/) via Ramachandran plot, and ProSA-web (https://prosa.services.came.sbg.ac.at/prosa.php) was used to detect potential structural anomalies. guinea pig L-ASNase (PDB 4R8L) and human arginase 1 (PDB 2ZAV) served as templates, and PyMOL (https://pymol.org/) was used for visualization. Substrate docking with AutoDock Vina^32^ was performed using l-asparagine and l-arginine, with binding affinities reported in kcal mol^–1^.

### 2.10 Statistical Analysis

Data are shown as mean ± SD (*n* = 3); significance was assessed by one-way ANOVA with Tukey/Dunnett post-tests (*p* < 0.05).

## 3 Results and Discussion

### 3.1 Recombinant Expression of 63_N_-h_C__hARG1

A dual-function chimeric enzyme (63_N_-h_C__hARG1), consisting of a hybrid l-asparaginase (63_N_-h_C_) fused to human arginase 1 (hARG1), was designed and expressed in different *E. coli* strains. SDS-PAGE analysis revealed that expression occurred exclusively as IBs (Figure 2), with no detectable soluble protein under any tested condition. IB-associated enzymatic assays confirmed the functionality of both enzymatic components (l-asparaginase and arginase) (Table 1). These assays were performed directly in the native extraction buffer (BugBuster, pH 8.0/37 °C), without protein quantification; therefore, specific activities could not be calculated.

**Figure 2:**
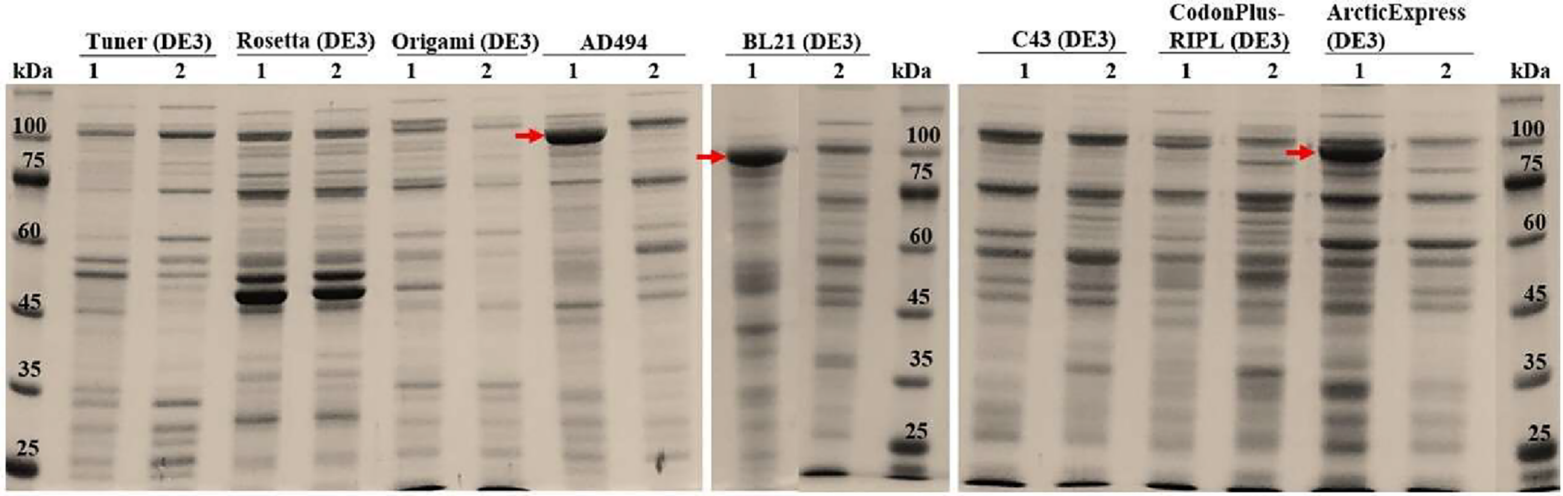
SDS-PAGE (10%) of 63_N_-h_C__hARG1 expression showing IB formation in *E. coli* *AD494, BL21 (DE3)* and *ArticExpress (DE3)* strains. Lysates from Lane 1 - cells transformed with 63_N_-h_C__hARG1/pET-22b­(+); Lane 2: cells with empty-vector control. Arrows indicate the expected ∼97 kDa chimera band. Molecular weight Marker: TrueColor High Range (10–245 kDa).

**Table 1:** Detectable Enzymatic Activities of 63_N_-h_C_ and hARG1 in Soluble and Insoluble Chimera Fractions Obtained from Various *E. coli* Strains

	**63**_**N**_ **-h**_**C**_ **enzymatic activity (U mL** ^**–1**^ **)** ^a^		**hARG1 enzymatic activity (U L** ^**–1**^ **)** ^a^	
**strain**	**soluble fraction**	**insoluble fraction**	**soluble fraction**	**insoluble fraction**
AD494	0.36 ± 0.02	1.38 ± 0.06	0.03 ± 0.04	7.68 ± 0.14
ArcticExpress (DE3)	0.07 ± 0.03	1.22 ± 0.11	0.07 ± 0.04	7.71 ± 0.13
BL21(DE3)	0.22 ± 0.05	1.20 ± 0.16	0.04 ± 0.02	7.23 ± 0.23
CodonPlus- RIPL (DE3)	0.22 ± 0.05	0.57 ± 0.05	0.12 ± 0.06	0.45 ± 0.08
C43(DE3)	0.21 ± 0.02	0.58 ± 0.06	0.11 ± 0.04	1.45 ± 0.10
Origami (DE3)	0.06 ± 0.01	0.24 ± 0.04	0.06 ± 0.04	0.09 ± 0.04
Rosetta (DE3)	0.36 ± 0.04	0.54 ± 0.04	0.06 ± 0.01	0.9 ± 0.10
Tuner (DE3)	0.08 ± 0.02	0.06 ± 0.03	0.08 ± 0.03	0.10 ± 0.01

a Activities expressed as U mL^–1^ for 63_N_-h_C_ and U L^–1^ for hARG1. Mean ± SD from triplicate assays; no statistical comparisons performed.

Expression was optimized by screening IPTG concentrations (0.01–1.0 mM) and induction temperatures (11–37 °C). The *pelB* leader sequence was included to target the protein to the periplasm, a more oxidizing environment expected to favor correct disulfide bond formation due to the high cysteine content (11 residues) of the chimera; however, none of the conditions yielded soluble 63_N_-h_C__hARG1, and *pelB* expression even decreased solubility, consistent with its limited efficiency for complex proteins.^33–35^ These results feature the difficulty of expressing large chimeric enzymes in prokaryotic hosts and the need for downstream recovery strategies.^36^ Because all conditions led to IB formation, optimal IB production was achieved with 0.01 mM IPTG at 20 °C for 16 h in *BL21­(DE3), AD494*, and *ArcticExpress­(DE3)* strains (Figure 3; Supplementary Figure S1). *BL21­(DE3)* was selected for subsequent solubilization and refolding due to its reliable yields and preservation of enzyme integrity.^37^

**Figure 3:**
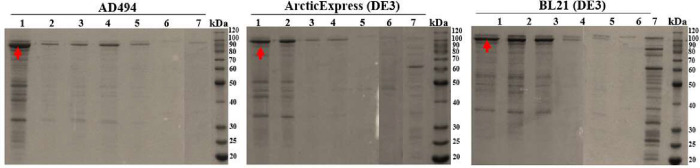
SDS-PAGE (10%) analysis showing the effects of varying IPTG concentrations and cultivation at 20 °C on the expression of 63_N_-h_C__hARG1 IBs. Lanes: lysates from cells transformed with 63_N_-h_C__hARG1/pET-22b­(+) and induced with IPTG at concentrations (mM): 1- 0.01; 2- 0.05; 3 −0.1; 4- 0.5; and 5- 1. Lane 6: lysate from cells with empty-vector control at 1 mM IPTG. Lane 7: lysate from noninduced culture. Arrows indicate the expected ∼97 kDa chimera band. Molecular weight marker: BenchMark Protein Ladder (10–220 kDa).

### 3.2 Obtaining the 63_N_-h_C__hARG1 Chimera from Nonclassical IBs

#### 3.2.1 Isolation

In the initial protein recovery phase, IBs were isolated from a 1 L *E. coli* culture pellet by lysozyme lysis followed by sonication. The IB pellet was sequentially washed to reduce nontarget proteins while maintaining high IB yield.^38^ Minimal protein loss was observed (Figure 4). The final suspension showed a strong SDS-PAGE band for the chimera, with a total protein concentration of 25.8 ± 0.6 mg mL^–1^. Despite aggregation, the chimera retained reproducible enzymatic activity in the IBs–associated form (2.16 ± 0.04 U mL^–1^ for 63_N_-h_C_ and 13.42 ± 0.09 U L^–1^ for hARG1), supporting the formation of nonclassical IBs that preserve partial native-like structure and function.^39,40^ In this study, measurable activity is defined as catalytic activity significantly above background levels and consistently detectable across independent assays. Direct comparison with purified parental enzymes under identical conditions was not performed; however, approximate activity ranges reported in previous kinetic studies of the individual enzymes suggest theoretical values of ∼19.2 U mL^–1^ for 63_N_-h_C_ ^12^ and ∼50 U L^–1^ for hARG1.^41^ Within this context, the activities observed for the chimera correspond to a partial retention of catalytic function, consistent with structural constraints imposed by fusion and oligomeric interference.

**Figure 4:**
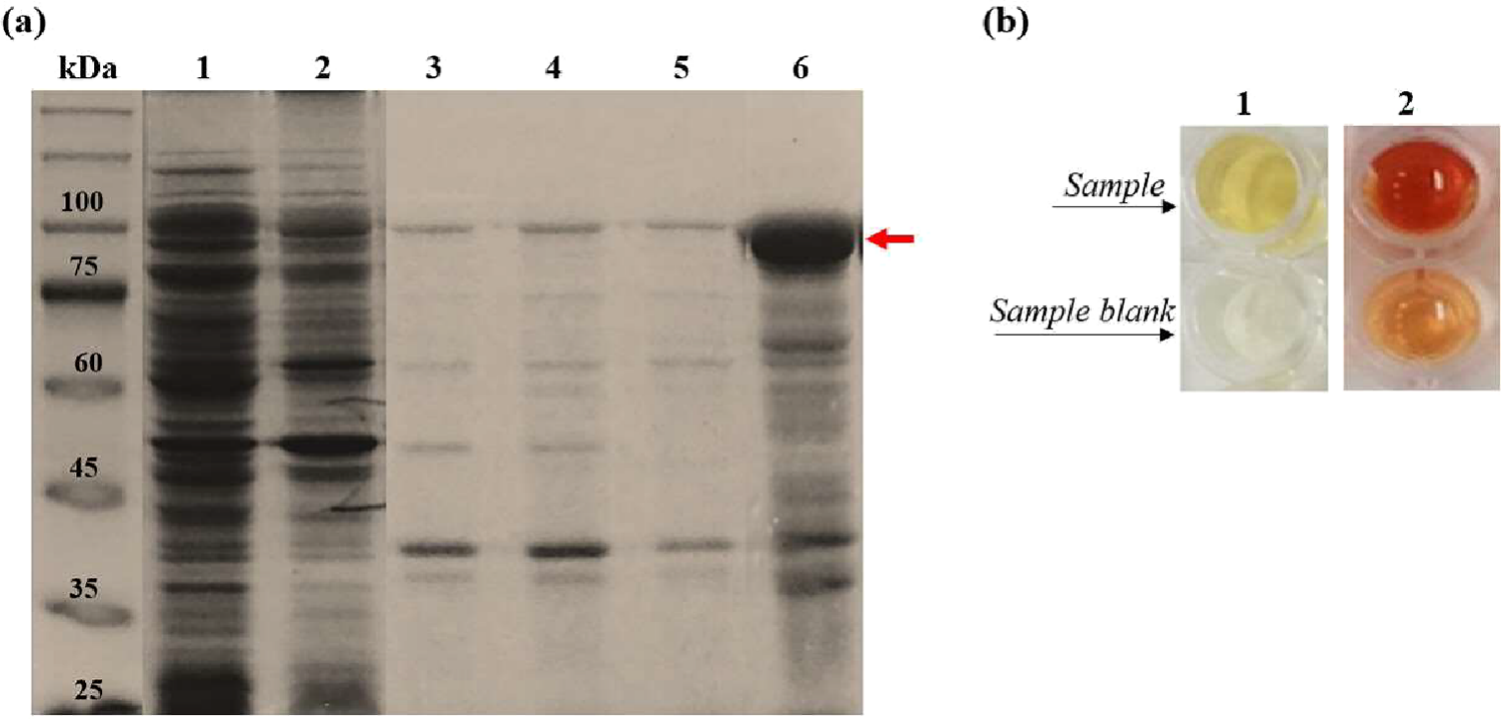
(a) SDS-PAGE (10%) of 63_N_-h_C__hARG1 IB isolation in *E. coli* *BL21­(DE3)* strain. Lanes: 1, soluble fraction; 2, supernatant obtained after washing the pellet with Triton X-100; 3–4, Tris-HCl buffer washes; 5, water wash; and 6, resuspended IBs in Tris-HCl (pH 7.4). Arrow indicates the expected ∼97 kDa chimera band. Molecular weight marker: TrueColor High Range Protein Marker (10–245 kDa). (b) Enzymatic activity detection: sample 1 L-ASNase (yellow-orange) by Nesslerization; sample 2 hARG1 (orange-red) by Arginase Assay Kit; both distinct from blanks.

#### 3.2.2 Solubilization

After IB isolation, solubilization was tested with urea (2, 4, 8 M) and GdnHCl (3, 6 M). Soluble and insoluble fractions were analyzed by SDS-PAGE, and efficiency (%) was calculated relative to total protein (∼1 mg mL^–1^). Highest recovery was achieved with 8 M urea (96.3%) and 6 M GdnHCl (94.4%), comparable to 2% SDS (99%) positive control. Lower concentrations (2–4 M urea) and 3 M GdnHCl showed poor solubilization (10–60%), similar to the Tris-HCl negative control (6.3%) (Figure 5a). Statistical analysis confirmed significant improvement with 8 M urea and 6 M GdnHCl versus control (*p* < 0.05) (Figure 5b). Based on overall performance and downstream compatibility, 8 M urea supplemented with 2-β-mercaptoethanol was selected as the optimal solubilization and refolding condition, as it produced the most favorable unfolded state for controlled refolding of the chimera. Fine-tuning the reducing environment was particularly critical for this construct, which contains 11 cysteine residues and therefore requires carefully regulated disulfide reshuffling to avoid mispairing and off-pathway folding. Collectively, these analyses indicate that 8 M urea provides sufficient denaturation strength while allowing a controlled redox environment conducive to productive refolding.^24^

**Figure 5:**
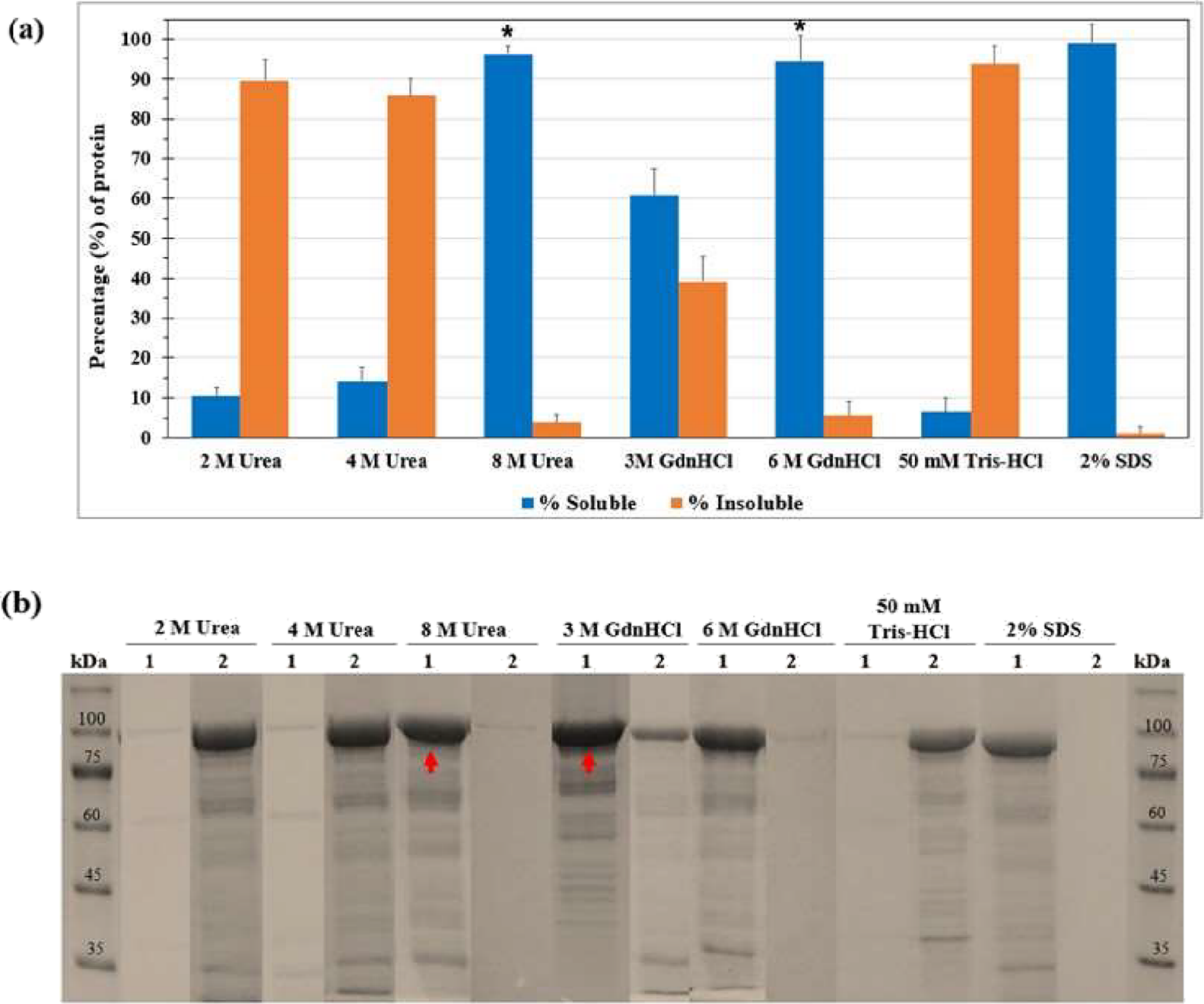
Solubilization efficiency of denaturing agents on 63_N_-h_C__hARG1 IBs. (a) Percentage comparison of soluble vs insoluble protein after treatment with different agents; Tris-HCl (pH 7.4) as negative control, 2% SDS as positive. Significant differences versus control: * (*p* < 0.05). Comparisons without notation indicate nonsignificance. (b) SDS-PAGE (10%) of soluble (Lanes 1) and insoluble fractions (Lanes 2). Red arrows mark conditions with highest solubilization. Molecular weight marker: TrueColor High Range Protein Marker (10–245 kDa).

#### 3.2.3 Purification of 63_N_-h_C__hARG1 by Gel Filtration and On-Column Refolding Methods

Because contaminant proteins can interfere with correct folding, IBs were purified by gel filtration. Based on the chromatographic profile (Figure 6a), fractions 7–13 contained a strong band at the expected molecular weight, with fractions 11 and 12 showing minimal impurities (Figure 6b). These were pooled, yielding ∼0.7 mg mL^–1^ of the chimera. This strategy effectively enriched the protein while maintaining its integrity, providing a clean starting point for refolding assays. As an alternative, the chimera was expressed with a C-terminal His-tag to allow Ni^2+^-affinity purification (Figure 6c). Although the protein bound to the column and migrated at ∼98 kDa on SDS-PAGE (Figure 6d), Western blot revealed extensive degradation, with most of the protein eluting as fragments (Figure 6e). This instability prevented its use in downstream experiments and highlighted a clear drawback compared with gel filtration, which preserved the full-length protein.

**Figure 6:**
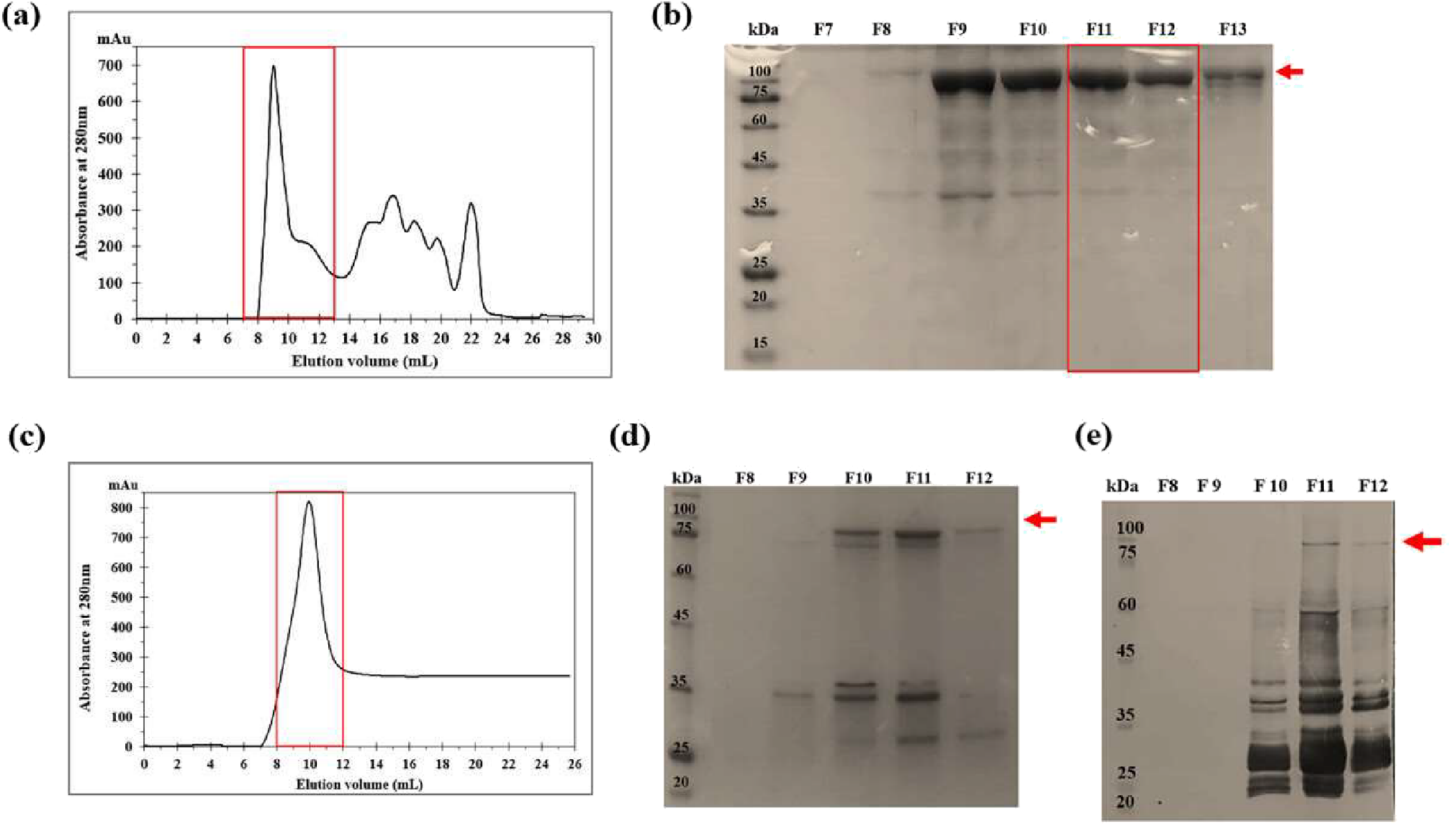
Comparison of two purification strategies for solubilized 63_N_-h_C__hARG1 IBs: size-exclusion chromatography (SEC) and on-column IMAC refolding. (a) SEC chromatogram; target protein eluted as a distinct peak (red rectangle). (b) SDS-PAGE analysis of SEC fractions F7 through F13. The ∼97 kDa chimeric protein is indicated by the red arrow. The selected fractions (F11–F12) are outlined in red. (c) IMAC chromatogram; solubilized IBs refolded and purified simultaneously, eluting as a peak (red). (d) SDS-PAGE of IMAC fractions (F8–F12) showing partial recovery (red arrow indicates ∼98 kDa chimera plus His-Tag). (e) Western blot of IMAC fractions confirming target protein with some degradation.

The His-tag also enabled testing of an on-column refolding strategy, coupling Ni^2+^-IMAC with solid-phase renaturation. A previous attempt using anion exchange chromatography was unsuccessful, because of the absence of detectable binding (data not shown). While conceptually attractive, the method consistently led to degradation of the chimera. The cysteine-rich 63_N_-h_C_ domain appeared particularly sensitive to oxidative stress during binding and elution, favoring aberrant disulfide bonds and irreversible modifications.^42^ These results indicate that IMAC-based refolding is not universally suitable and that protein-specific chemical properties must be carefully considered. Gel filtration offered a gentler purification approach, preserving protein integrity, whereas IMAC on-column refolding extensive caused degradation, likely due to harsher conditions.

#### 3.2.4 Effect of Additives on Aggregation and Refolding Strategies

During refolding, unfolded intermediates are prone to aggregation due to exposed hydrophobic regions, reducing efficiency and yield.^43,44^ To address this, several additives were tested after solubilization in 8 M urea and gel filtration. Turbidity measurements (OD_320_ nm) showed that only 200 mM fructose significantly decreased aggregation versus control (*p* < 0.05), indicating a protective osmolyte effect. Glycerol, sucrose, and glutamic acid had little impact, while dextran increased aggregation (Figure 7). Full data and statistical analyses are provided in the Supporting Information. These results emphasize the importance of additive selection, with simple sugars like fructose improving solubility and reducing aggregation during refolding.

**Figure 7:**
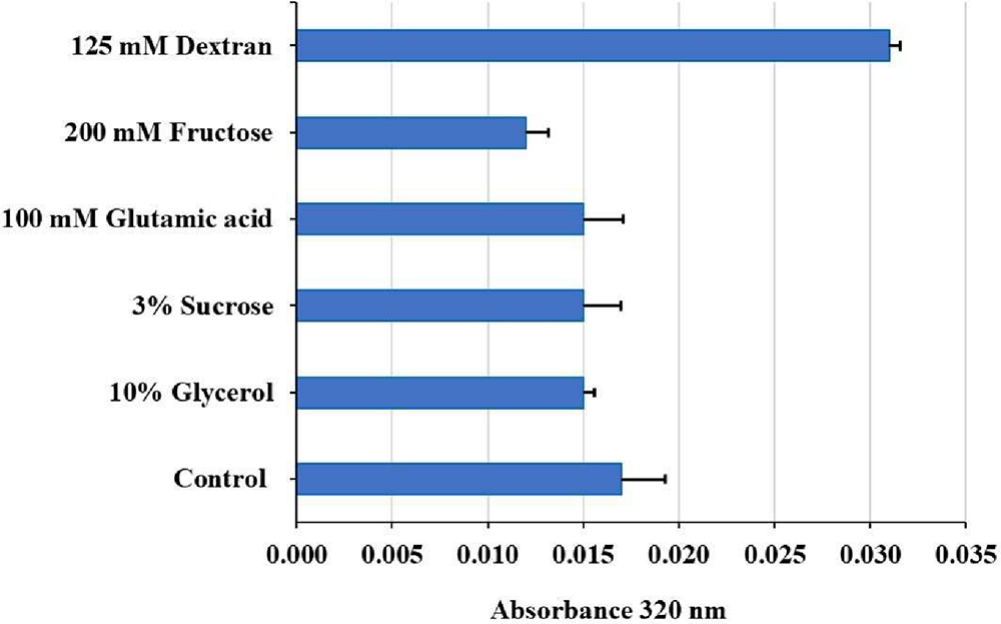
Effect of refolding additives on 63_N_-h_C__hARG1 chimera disaggregation, after dilution in refolding buffer with 0.5 M urea. Lower absorbance indicates higher disaggregation. Error bars: mean ± SD (*n* = 3); * (*p* < 0.05) versus control. Comparisons without notation indicate nonsignificance.

The optimized formulation (Tris-HCl, NaCl, 0.5 M urea, 200 mM fructose, 2-β-mercaptoethanol) was used in reverse dilution, drip, and stepwise dialysis. Both dilution methods improved chimera-specific activities versus solubilized IBs, with reverse dilution achieving slightly higher purification folds (Table 2). Recovery of both l-asparaginase and arginase 1 activities was observed, though precipitation during dialysis reduced yields, indicating that buffer alone cannot fully prevent aggregation. Enzymatic activities were measured at 37 °C in Tris-HCl (50 mM, pH 7.4) at pH 8.8 for 63_N_-h_C_ and pH 9.5 for hARG1. Testing multiple refolding strategies revealed that dilution-based approaches were the most effective for the chimeric enzyme. Dilution-based refolding methods have been successfully applied to renature L-ASNase from IBs.^45^ While some enzymatic activity was restored, full recovery was not achieved, indicating persistent folding challenges. Precipitation during dialysis indicated that, despite its common use, this approach was suboptimal for this chimera, likely because the slow diffusion of denaturant offers poor control over refolding kinetics and promotes aggregation.^46^ These results emphasize that dilution-based methods, supported by selective additives, provide a workable platform but require further optimization to achieve higher refolding efficiency and stability.

**Table 2:** Comparison of l-Asparaginase and Human Arginase 1 Activities, and Purification Fold for Solubilized IBs and Refolded Samples of the 63_N_-h_C__hARG1 Chimera

		**63**_**N**_ **-h**_**C**_ ^a^			**hARG1** ^a^		
**sample**	**protein concentration (mg mL^–1^)**	**enzymatic activity** **(U mL** ^**–1**^)	**specific activity** **(U mg** ^**–1**^)	**purification factor (fold)**	**enzymatic activity** **(U L** ^**–1**^)	**specific activity** **(U mg** ^**‑1**^)	**purification factor (fold)**
isolated IBs	25.8 ± 0.6	2.16 ± 0.04	0.084 ± 0.0025	1	13.42 ± 0.09	(5.20 ± 0.013) × 10^–4^	1
refolding by drip dilution	1^b^	0.16 ± 0.04	0.16 ± 0.04	1.91 ± 0.48	4.35 ± 1.1	(4.35 ± 0.11) × 10^–3^	8.36 ± 2.12
refolding by reverse dilution	1^b^	0.22 ± 0.05	0.22 ± 0.05	2.63 ± 0.60	4.66 ± 0.9	(4.66 ± 0.09) × 10^–3^	8.96 ± 1.74

a Mean ± SD from triplicate assays.

b Protein concentration in dilution experiments was maintained at ∼1 mg mL^–1^.

### 3.3 Bioinformatic Evaluation

#### 3.3.1 Physicochemical Parameters

ProtParam analysis (Table 3) indicated that 63_N_-h_C__hARG1 has favorable stability (instability index <40), mild hydrophobicity (positive GRAVY), a moderately acidic pI (5.89), consistent with its relatively low abundance of basic residues (Arg + Lys = 85), and high aliphatic index (>80), suggesting thermostability. Protein-Sol predicted poor solubility (score 0.343), consistent with IBs formation in *E. coli*.^47^ The raw Protein-Sol outputs are presented in Supplementary Figure S2. Eleven cysteine residues allowed prediction of four disulfide bonds, including two long-range interactions (Cys173–Cys198, Cys564–Cys628) likely stabilizing interdomain structure, supporting a compact and stable tertiary fold despite the artificial fusion (Table 4).

**Table 3:** Physicochemical Evaluation of Construction of 63_N_-h_C__hARG1

**parameter**	**result**
number of amino acids	905
theoretical isoelectric point (pI)	5.89
molecular weight (MW) (kDa)	96.89
instability index	32.36
aliphatic index	101.09
total number of positively charged residues (Arg+Lys)	85
total number of negatively charged residues (Asp+Glu)	99
amino acid cysteine % composition	1.2% (11/905)
grand average of hydropathicity (GRAVY)	0.012

**Table 4:** Predicted Disulfide Bond Pairs (Cysteine (Cys) Position), Ranked in Descending Order of Probability

**bond index**	**Cys1 position**	**Cys2 position**
1	173	198
2	564	628
3	296	299
4	404	504

#### 3.3.2 Secondary Structure and Homology Modeling

GOR IV predicted 63_N_-h_C__hARG1 to contain 35.4% α-helices, 16.0% β-sheets, and 48.6% random coils, indicating a mainly α-helical yet flexible architecture, with high coil content reflecting the interdomain linker and modular design (Supplementary Figure S3). AlphaFold2 homology modeling generated five structures; the top model had a near-perfect pLDDT score, high Ramachandran statistics (94.7% favored and 96.8% allowed regions), and a ProSA Z-score of – 14.74, fell within the range of experimentally determined high-quality protein structures^48,49^ (Figure 8; Supplementary Figure S4). Although ∼3.2% of residues were flagged as outliers in the Ramachandran plot, these deviations are minor and likely localized to flexible regions, not compromising the overall fold. Collectively, the data support that the chimera adopts a stable 3D structure compatible with its dual-domain design.

**Figure 8:**
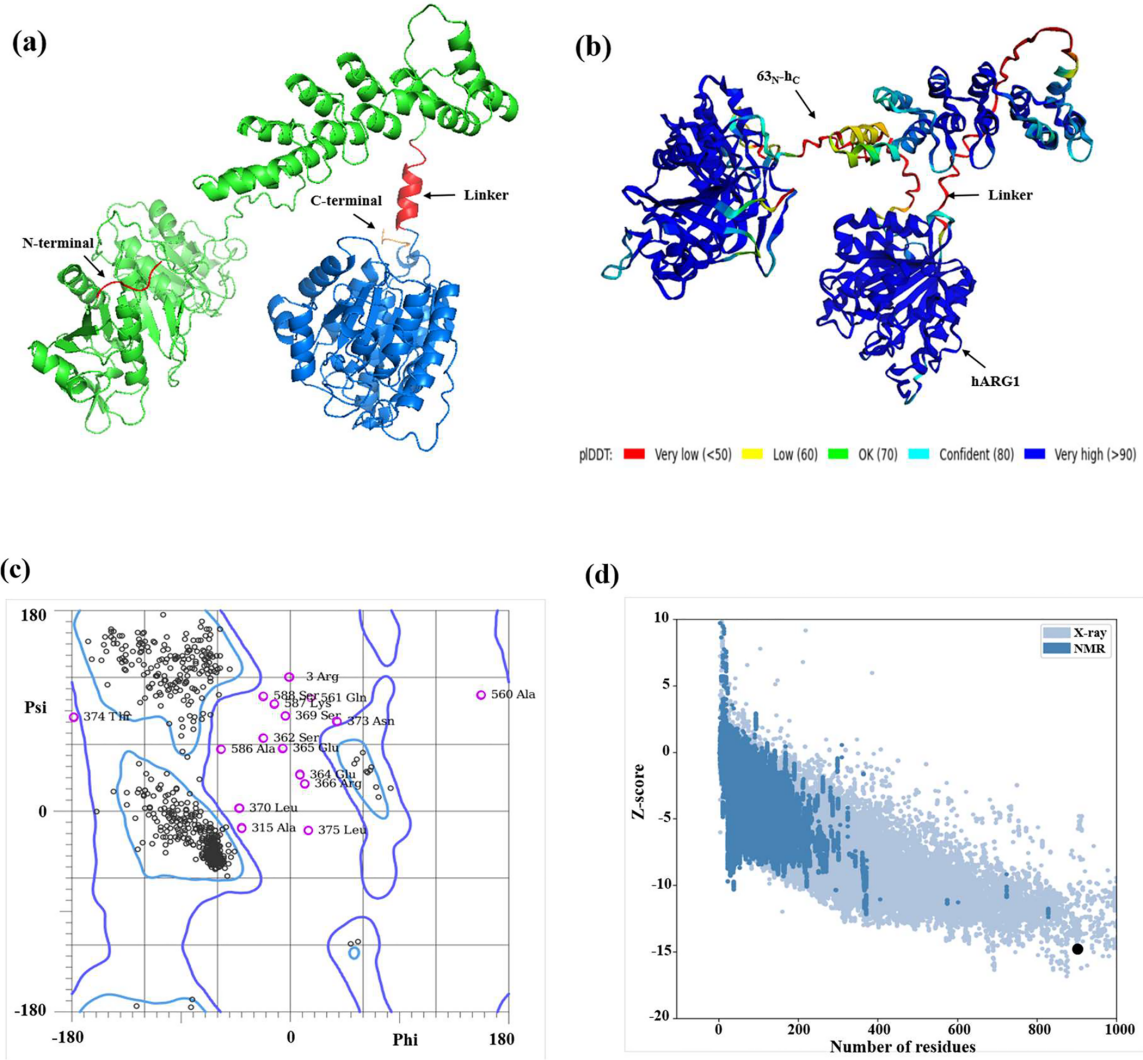
Monomeric model of 63_N_-h_C__hARG1 chimeric enzyme, 3D modeling and validation. (a) Schematic structural representation of the parental domains used to generate the chimera, showing representative monomeric models of 63_N_-h_C_ (green) and hARG1 (blue) connected by the rigid α-helical linker (red). (b) AlphaFold2-predicted monomer, colored by pLDDT confidence (blue = high, red = low). Residues are not labeled, as this representation aims to illustrate the overall monomer organization and folding. (c) Ramachandran plot showing the distribution of backbone dihedral angles: black circles, favored; allowed, between contours; magenta, outliers (e.g., Arg3, Ala315, Ala560). (d) ProSA Z-score of – 14.74 (black dot), consistent with high-quality native-like structure.

#### 3.3.3 Molecular Docking Simulation

Molecular docking indicated lower predicted binding affinities for 63_N_-h_C__hARG1 compared with the native enzymes (−4.9 vs −7.58 kcal mol^–1^ for 63_N_-h_C_; −5.3 vs −6.9 kcal mol^–1^ for hARG1), likely due to conformational effects of domain fusion. Despite this, results aligned with enzymatic assays, confirming retention of both l-asparaginase and arginase 1 catalytic activities.

### 3.4 Final Considerations

Designing multimeric chimeric proteins poses substantial challenges due to the requirement for precise quaternary assembly. Unlike monomeric fusions, multimeric chimeras are particularly prone to misassembly, aggregation, and reduced catalytic efficiency. Linker design plays a critical role in mitigating these issues: flexible Gly/Ser-rich linkers enable domain mobility,^26^ whereas rigid α-helical motifs such as A­(EAAAK)­nA maintain spatial separation and reduce steric interference.^27,50,51^ In the case of 63_N_-h_C__hARG1, a rigid helical linker [A­(EAAAK)_2_A] was selected to stabilize domain orientation. However, linker rigidity alone cannot fully resolve structural incompatibilities, as 63_N_-h_C_ and hARG1 naturally assemble as tetramers and trimers, respectively.^15,52,53^ Their forced coexistence within a single polypeptide chain may promote heterogeneous oligomerization, altered stoichiometry, or partial functional impairment.^54^ Misassembly can disrupt folding and reduce activity, whereas heterogeneous oligomerization may yield inactive complexes and aggregates. Furthermore, the expression of large multidomain proteins in prokaryotes often results in misfolding and low yields.^55,56^

Despite these intrinsic structural constraints, the chimera retained measurable enzymatic activity in both domains, indicating the formation of at least partially functional oligomeric assemblies. Two conceptual oligomerization scenarios can be considered: (i) independent assembly of the 63_N_-h_C_ and hARG1 domains into tetrameric and trimeric states, respectively, which is sterically unfavorable within a fused construct; or (ii) forced assembly into a single, non-native but functionally permissive oligomeric state imposed by the fusion architecture.

Based on the molecular weights of the parental enzymes (61.1 kDa for 63_N_-h_C_ ^12^ and 34.7 kDa for hARG1 ^57^) and the estimated molecular weight of the chimera, the formation of a high-molecular-weight multimeric complex (approximately 290–390 kDa) is plausible; this type of large construct remains relatively rare.^58,59^ However, direct experimental determination of the oligomeric state (e.g., by DLS or SEC–MALS) was not performed in this study and therefore represents a limitation. Accordingly, the oligomerization model proposed here should be regarded as putative and intended to provide a conceptual framework for interpreting the observed partial activity rather than a definitive structural assignment.

The reduced enzymatic activity observed after refolding compared to the IBs state can be explained by several structural and biochemical factors. Nonclassical IBs are known to retain partially ordered conformations that preserve residual catalytic function. Upon solubilization with strong denaturants, these conformations are disrupted, and recovery of the fully active state depends on the efficiency of folding pathways and multimeric assembly. In the case of 63_N_-h_C__hARG1, both domains require correct oligomerization (tetrameric for L-ASNase and trimeric for hARG1) and, in the case of hARG1, proper metal coordination. Also, the chimera contains 11 cysteine residues, making it prone to disulfide mispairing during refolding, especially under partially reducing conditions. In this study, no exogenous Mn^2+^ was included in the refolding or assay buffers; nevertheless, measurable hARG1 activity was detected, consistent with reports showing residual function in partially metal-depleted preparations. Whether controlled Mn^2+^ supplementation could enhance refolding efficiency or catalytic recovery remains a question for future work.

During refolding, kinetic competition with off-pathway aggregation, incomplete quaternary assembly, and misfolding events reduce the proportion of correctly folded species. As a result, although refolding strategies restored measurable activity, they did not reach the levels observed in IB-associated forms. These findings accentuate the intrinsic challenges of recovering full activity from IBs and emphasize the need for further optimization of refolding additives and stabilization protocols.

## 4 Conclusions

This study reports the design, recombinant expression, and functional recovery of a novel bifunctional chimeric enzyme, 63_N_-h_C__hARG1, combining l-asparaginase and human arginase 1. Expression in *E. coli* resulted predominantly in IBs that retained measurable enzymatic activity, highlighting the relevance of nonclassical IBs as reservoirs of partially folded and functional protein species. Although the fusion of those two enzymes, with distinct oligomeric architectures, imposes significant structural and folding challenges, the recovery of activity in both domains demonstrates the feasibility of producing complex multimeric chimeras using bacterial hosts. The systematic evaluation of expression, solubilization, purification, and refolding conditions presented here provides a practical workflow for handling aggregation-prone, multimeric fusion proteins. While the exact oligomeric state of the chimera remains to be experimentally defined, the results support a model in which partial native-like folding and non-native but functionally permissive assemblies enable residual catalytic activity. Importantly, the limitations identified, particularly those related to oligomerization constraints, disulfide reshuffling, and refolding efficiency, offer valuable guidance for future optimization and biophysical characterization. Overall, this work contributes methodological insights into the preparative production and recovery of multimeric chimeric enzymes from IBs and supports the development of scalable strategies relevant to enzyme engineering, bioprocess development, and industrial biotechnology.

## Supplementary Material



## Data Availability

All data supporting the findings of this study are available within the article and its Supporting Information.

## Notes

Amino acid sequence of the 63N-hc_hARG1 chimera; characteristics of *E. coli* strains; SDS-PAGE optimization analysis; protein-Sol predicted solubility data; GOR IV secondary structure prediction data; and AlphaFold2 structural models (PDF)
