# Supplementary material for: Expression, Solubilization, and Refolding Recovery of a Novel l‑Asparaginase–Arginase 1 Chimera from E. coli Inclusion Bodies
Source: ACS Omega. 2026 Mar 6;11(10):16333–43. doi: 10.1021/acsomega.5c12075 (PMC13000580; doi:10.1021/acsomega.5c12075)

Supporting Information

Expression, Solubilization, and Refolding Recovery of a Novel L-Asparaginase–Arginase 1 Chimera from *E. coli* Inclusion Bodies

Massiel V. Rivera [a], Marina Gabriel Fontes [a], William Henry Roldán [a], Roberto Carlos Vieira da Silva Junior [a], Lisandra Herrera Belén [b], Jorge F. Beltrán [c], Igor Lopes-Silva [a], Adalberto Pessoa Jr. [a], Marco A. Stephano [a], Jorge G. Farias [c], Tales Alexandre Costa-Silva [d] and Gisele Monteiro [a*]

* E-mail: smgisele@usp.br

[a] Departamento de Tecnologia Bioquímica-Farmacêutica, Faculdade de Ciências Farmacêuticas, Universidade de São Paulo, São Paulo, 05508-000, Brazil.

[b] Departamento de Ciencias Básicas, Facultad de Ciencias, Universidad Santo Tomás, Temuco, 4780000, Chile.

[c] Departamento de Ingeniería Química, Facultad de Ingeniería y Ciencias, Universidad de La Frontera, Temuco, 4811230, Chile.

[d] Center for Natural and Human Sciences, Federal University of ABC, Santo André, São Paulo, 09280-560, Brazil.

Table of contents

Text S1: Amino acid sequence of 63_N-h_C_hARG1 chimera.

Table S1. Characteristics of the *E. coli* strains used in this study.

Figure S1: SDS-PAGE (10%) analysis results for the optimization assessment of the influence of IPTG induction levels and cultivation temperatures on the expression of recombinant 63_N-h_C_hARG1 in *E. coli* host strains.

Figure S2: raw data generated by the Protein-Sol server for predicted scaled solubility.

Figure S3: raw data generated by the GOR IV server for secondary structure prediction.

Figure S4: Raw data generated by AlphaFold2 software for the generated models for 63_N-h_C_hARG1.

Text S1: Amino acid sequence of 63_N-h_C_hARG1 chimera.

```
        10         20         30         40         50         60
MARASGSERH LLLIYTGGTL GMQSKGGVLV PGPGLVTLLR TLPMFHDKEF ARARGLSEDT

        70         80         90        100        110        120
LVLPPASRNQ RILYTVLECQ PLLDSSDMTI DDWIRIAKII ERHYEQYQGF VVIHGTDTMA

       130        140        150        160        170        180
FAASMLSFML ENLHKPVILT GAQVPIRVLW NDARENLLGA LLVAGQYIIP EVCLFMNSQL

       190        200        210        220        230        240
FRGNRVTKVD SQKFEAFCSP NLSPLATVGA DVTIAWDLVR KVKWKDPLVV HSNMEHDVAL

       250        260        270        280        290        300
LRLYPGIPAS LVRAFLQPPL KGVVLETFGS GNGPSKPDLL QELRAAAQRG LIMVNCSQCL

       310        320        330        340        350        360
RGSVTPGYAT SLAGANIVSG LDMTSEAALA KLSYVLGLPE LSLERRQELL AKDLRGEMTP

       370        380        390        400        410        420
PSVEERRPSL QGNTLGGGVS WLLSLSGSQE ADALRNALVP SLACAAAHAG DVEALQALVE

       430        440        450        460        470        480
LGSDLGLVDF NGQTPLHAAA RGGHTEAVTM LLQRGVDVNT RDTDGFSPLL LAVRGRHPGV

       490        500        510        520        530        540
IGLLREAGAS LSTQELEEAG TELCRLAYRA DLEGLQVWWQ AGADLGQPGY DGHSALHVAE

       550        560        570        580        590        600
AAGNLAVVAF LQSLEGAVGA QAPCPEVLPG VAEAAAKEAA AKAMSAKSRT IGIIGAPFSK

       610        620        630        640        650        660
GQPRGGVEEG PTVLRKAGLL EKLKEQECDV KDYGDLPFAD IPNDSPFQIV KNPRSVGKAS

       670        680        690        700        710        720
EQLAGKVAEV KKNGRISLVL GGDHSLAIGS ISGHARVHPD LGVIWVDAHT DINTPLTTTS

       730        740        750        760        770        780
GNLHGQPVSF LLKELKGKIP DVPGFSWVTP CISAKDIVYI GLRDVDPGEH YILKTLGIKY

       790        800        810        820        830        840
FSMTEVDRLG IGKVMEETLS YLLGRKKRPI HLSFDVDGLD PSFTPATGTP VVGGLTYREG

       850        860        870        880        890        900
LYITEEIYKT GLLSGLDIME VNPSLGKTPE EVTRTVNTAV AITLACFGLA REGNHKPIDY

LNPPK
```

Blue: 63_N-h_C, 571 amino acids,
Orange: *linker*, 12 amino acids,
Green: hARG1, 322 amino acids.
Total: 905 amino acids.

40

Table S1. Characteristics of the *E. coli* strains used in this study.

Strain	Genotype	Selection marker and requirement	Source
ArcticExpress (DE3)[1]	[F$^-$ *ompT hsdS* (r_B^- m_B^-) *dcm*$^+$ Tet^r *gal* λ (DE3) *endA* Hte [*cpn10 cpn60* Gent^r]]	20 µg mL^{-1} Gent[10]	Agilent Technologies ™
AD494[2]	*[Δ (araABC-leu) 7697 ΔlacX74 ΔmalF3 ΔphoAPvull phoR trxB ::Kan^{r}F' [lacI^q lacZΔM15proAB$^+$]]*	15 µg mL^{-1} Kan	Novagen ™
BL21(DE3)[3]	[*F$^-$, omp T, hsdSb (r_B^- m_B^-), gal, dcm (DE3)*]	-	Life Technologies ™
CodonPlus-R4IPL (DE3)[4]	*[F$^-$ ompT hsdS (r_B^- m_B^-) dcm$^+$ Tet^r gal λ(DE3) endA Hte [argU proL Cam^r] [argU ileY leuW Strep/Spec^r]*	34 µg mL^{-1} Cam, 50 µg mL^{-1} Strep e 12.5 µg mL^{-1} Tetra	Agilent Technologies ™
C43 (DE3)[5]	[F$^-$*ompT gal hsdSB* ($r_B^-$$m_B^-$) gal *dcm, (*DE3)]	-	Lucigen ™
DH5α[6]	*[endA1, hsdR17 (rk$^-$ mk$^+$), supE44, thi-1, recA1, gyrA (Na 1r), relA1, Δ(lacZYA-argF) $_{U169}$ (Φ80lacZΔM15)]*	-	Invitrogen ™
Origami (DE3)[7]	*[1Δ(ara$^-$leu)7697 ΔlacX74 ΔphoA PvuII phoR araD139 ahpC galE galK rpsL F'[lac$^+$ lacI q pro] (DE3) gor522::Tn10 trxB (Kan^r, Str^r, Tet^r)]*	15 Kan µg mL^{-1}, 50 µg mL^{-1} Strep e 12.5 µg mL^{-1} Tetra	Novagen ™
Rosetta (DE3)[8]	*[F$^-$ ompT hsdSB (r_B^- m_B^-) gal dcm (DE3) pRARE2 (Cam^r)]*	34 µg mL^{-1} Cam	Novagen ™
Tuner (DE3)[9]	*[F$^-$ ompT hsdSB ($r_B^-$$m_B^-$) dcm, gal lacY1 (DE3)]*	-	Novagen ™

- = no selection marker, Kan = kanamycin, Cam = chloramphenicol, Gent=geneticin, Tetra = tetracycline, Strep = streptomycin.
[1]. This system expresses chaperone proteins to increase the solubility of insoluble proteins. [2]. Strain with a mutation in *trxB*, which allows the formation of disulfide bridges in proteins in the *E. coli* cytoplasm. [3]. It is deficient in Lon and OmpT proteases, making it useful for the expression of cytoplasmic proteins. [4]. It contains extra copies of genes for rare tRNAs, facilitating the translation of heterologous proteins and enabling high expression of recombinant genes. [5]. This host is effective for the expression of toxic and membrane proteins. [6]. It is suitable for cloning and storage of expression vectors. [7]. The *trxB/gor* mutant promotes the efficient formation of disulfide bonds in the cytoplasm. [8]. It is optimized to express eukaryotic proteins that use rare tRNA codons in *E. coli*. [9]. The *lacZY* mutant allows adjustable control of protein expression throughout the bacterial culture. [10]. To produce ArcticExpress (DE3) electrocompetent cells, 10 µg mL^{-1} of tetracycline should be added.

Figure S1. SDS-PAGE (10%) showing the effect of IPTG concentration and induction temperature on 63_N-h_C_hARG1 expression in *E. coli* strains (a) AD494, (b) ArcticExpress(DE3), and (c) BL21(DE3). Lanes: cells transformed with 63_N-h_C_hARG1/pET-22b(+) and induced with IPTG at concentrations (mM): 1- 0.01; 2- 0.05; 3 -0.1; 4- 0.5; and 5- 1. Lane 6: cells with empty-vector control at 1 mM IPTG. Lane 7: Non-induced culture. Red arrow indicates the ~97 kDa chimera band. Molecular marker: BenchMark™ Protein Ladder (10-220 kDa).

(a)

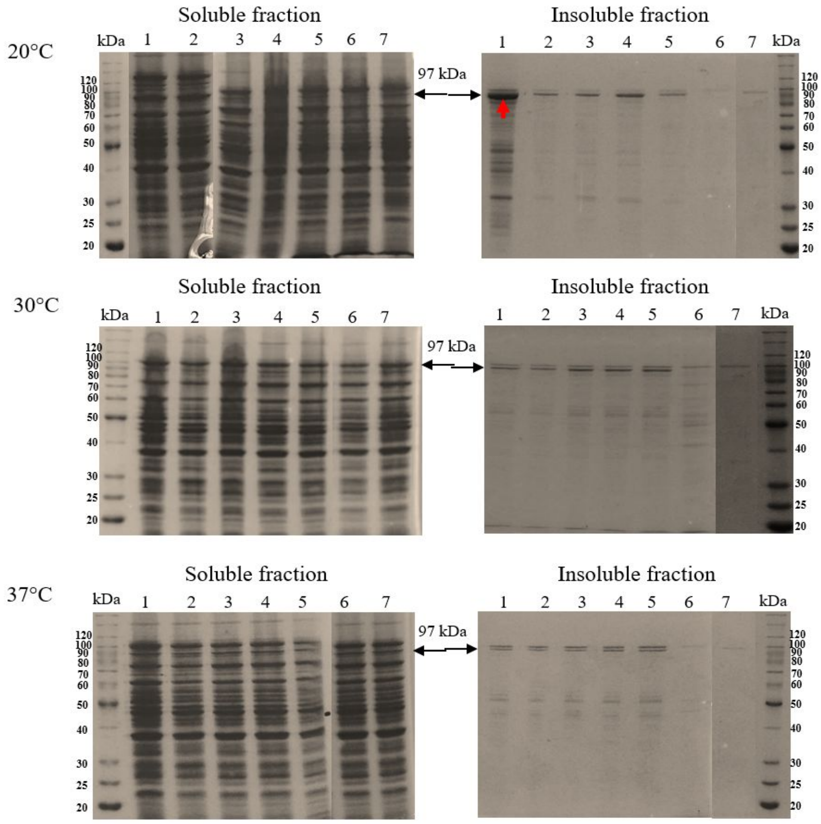

Figure S1: (continuation)

(b)

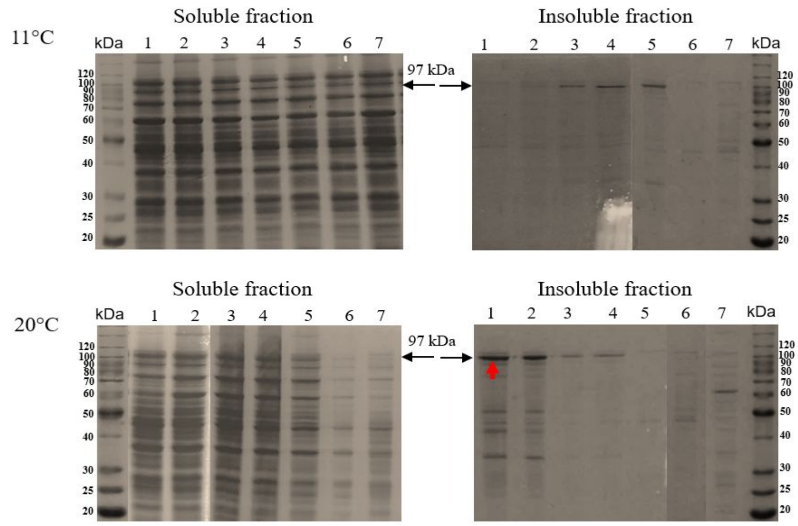

(c)

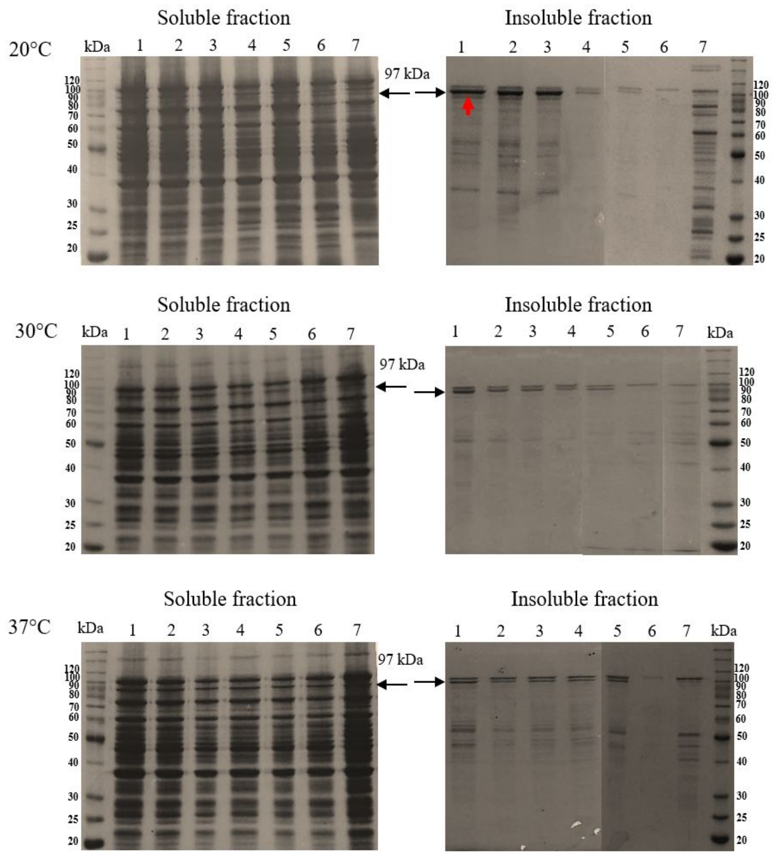

Figure S2: raw data generated by the Protein-Sol server for predicted scaled solubility.

1- The predicted solubility (QuerySol) for 63_N-h_C_hARG1 chimera is 0.34, which is below the population average of 0.45. This indicates that the protein is predicted to be less soluble than the average soluble *E. coli* proteins in the experimental dataset.

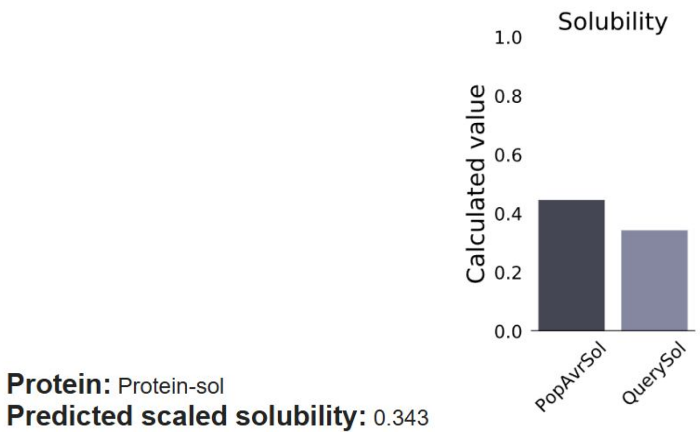

2- Deviations from population average: yellow bars represent positive deviations, indicating that certain residues or properties are more abundant in the chimera compared to the average soluble proteins. Green bars show negative deviations, meaning those elements are less represented. Significant deviations—whether positive or negative—can impact the chimera's solubility.

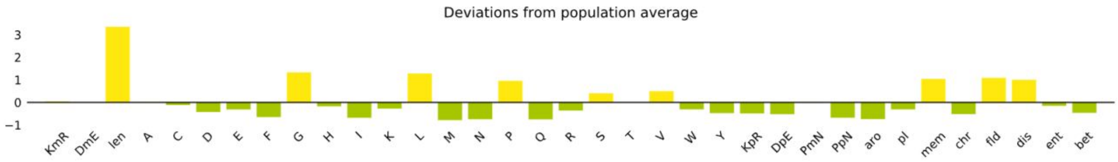

Figure S2: (continuation)

3-Windowed charge score per amino acid: This plot highlights local charge variations along the primary sequence. Red bars indicate regions with a net negative charge, which may be associated with aggregation or solubility issues. In contrast, blue bars reflect neutral or positively charged regions, which are typically more favorable for solubility.

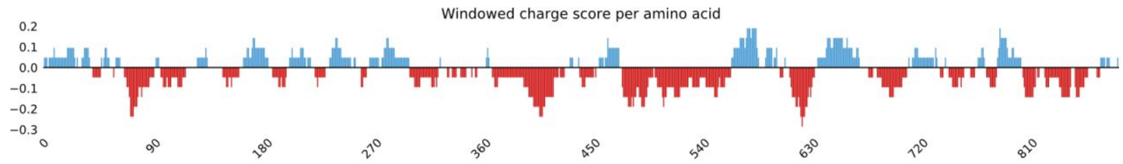

4-Windowed fold propensity per amino acid: this panel reflects the predicted folding tendency across the sequence. Orange bars denote regions with high fold propensity, suggesting structured and potentially stable areas. Blue bars point to low fold propensity, often corresponding to disordered or flexible regions that may affect solubility and promote aggregation.

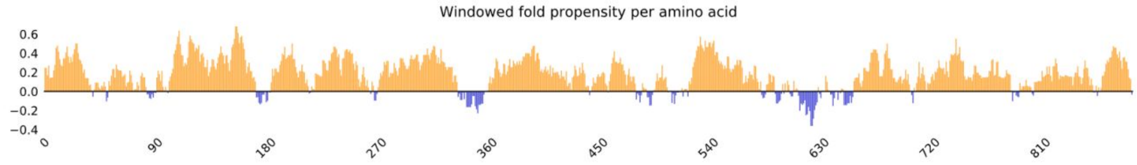

Figure S3: raw data generated by the GOR IV server for secondary structure prediction.

1- Textual alignment view (left) and text summary (right): protein sequence (one-letter amino acid code in black). Predicted secondary structure elements below each amino acid: α-helix (**h** in blue); β-sheets (**e** in red); random coil/ no regular secondary structure (**c** in orange). _

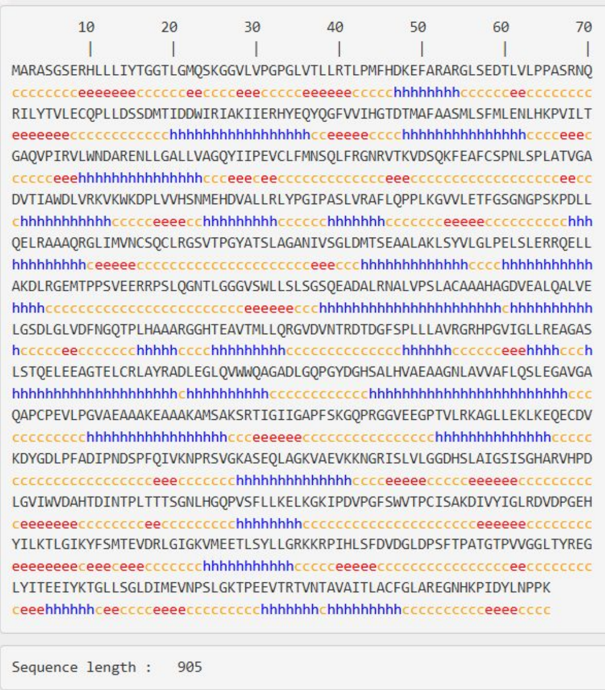

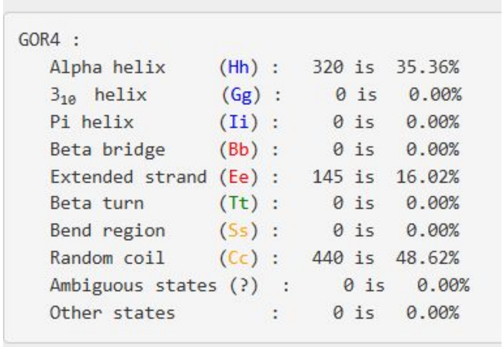

2-Bar graph: horizontal bar plot shows the location of secondary structure elements along the 63_N-h_C_hARG1 sequence, likely representing α-helices (red bars), β-sheets (blue bars), coils or unstructured regions (white gaps). α- helix and β-sheets are distributed along the sequence. The structure appears to alternate frequently between coils, helices, and sheets.

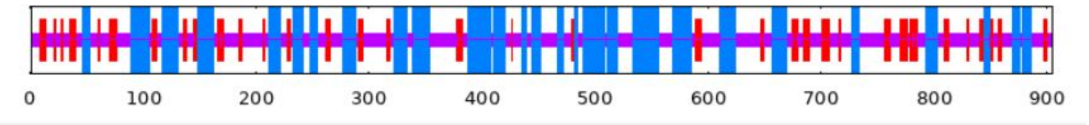

Figure S3: (continuation)

3-Probability curves: probabilities of each residue being in a given secondary structure type: Blue line, probability of α- helices; red line, probability of β-sheets; magenta/pink line, probability of coil.

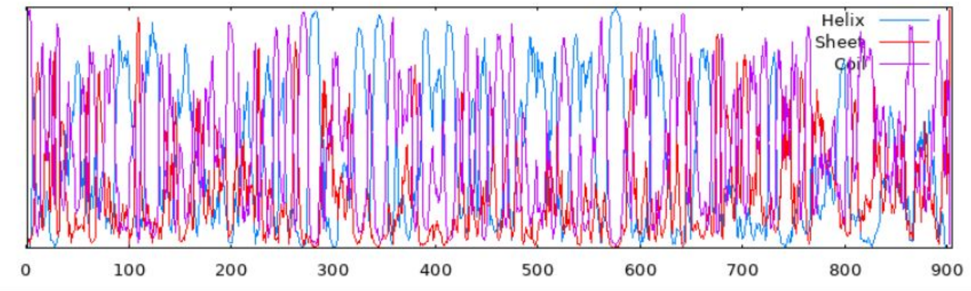

Figure S4: Raw data generated by AlphaFold2 software for the generated models for 63_N-h_C_hARG1.

1- PAE (Predicted Aligned Error) matrix for five models ranked by confidence. The matrix helps evaluate structural confidence and domain flexibility, as well as measure uncertainty in the relative positioning of different protein regions. The color scale indicates: blue (low PAE, 0–10) means high confidence in spatial positioning, while red (high PAE, >20–30) indicates low confidence, suggesting uncertainty in relative positioning. The X and Y axes represent the protein residues.

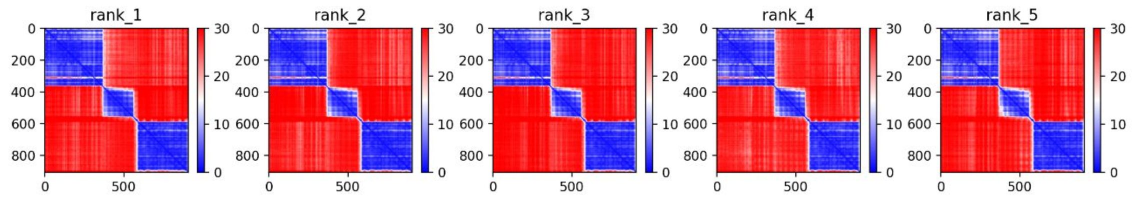

2- Coverage plot**,** based on similar sequences found (Y-axis) and the positions of amino acid residues in the sequences (X-axis). On the side, there is a vertical colored bar indicating the sequence identity by similarity with the input sequence.

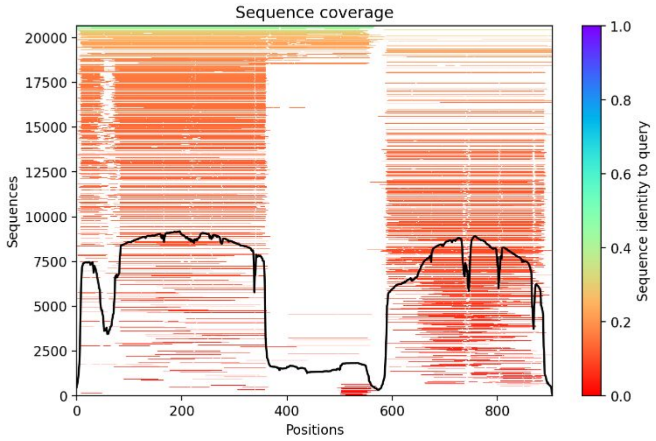

Figure S4: (continuation)

3- pLDDT (predicted local distance difference test) prediction by position for the five structural models of the sequence that were generated and ranked (rank 1 to 5).

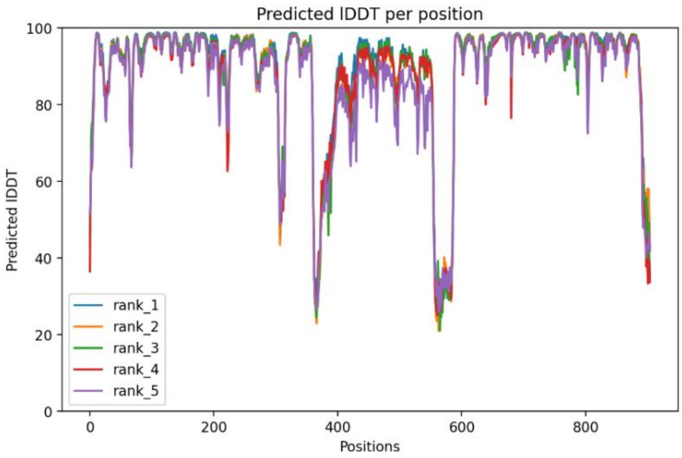

Supplement: Supplementary file 1 [file ao5c12075_si_001.pdf]
